# Supplementary figures and images for: NRP2 as an Emerging Angiogenic Player; Promoting Endothelial Cell Adhesion and Migration by Regulating Recycling of α5 Integrin
Source: Front Cell Dev Biol. 2020 May 26;8:395. doi: 10.3389/fcell.2020.00395 (PMC7264094; doi:10.3389/fcell.2020.00395)

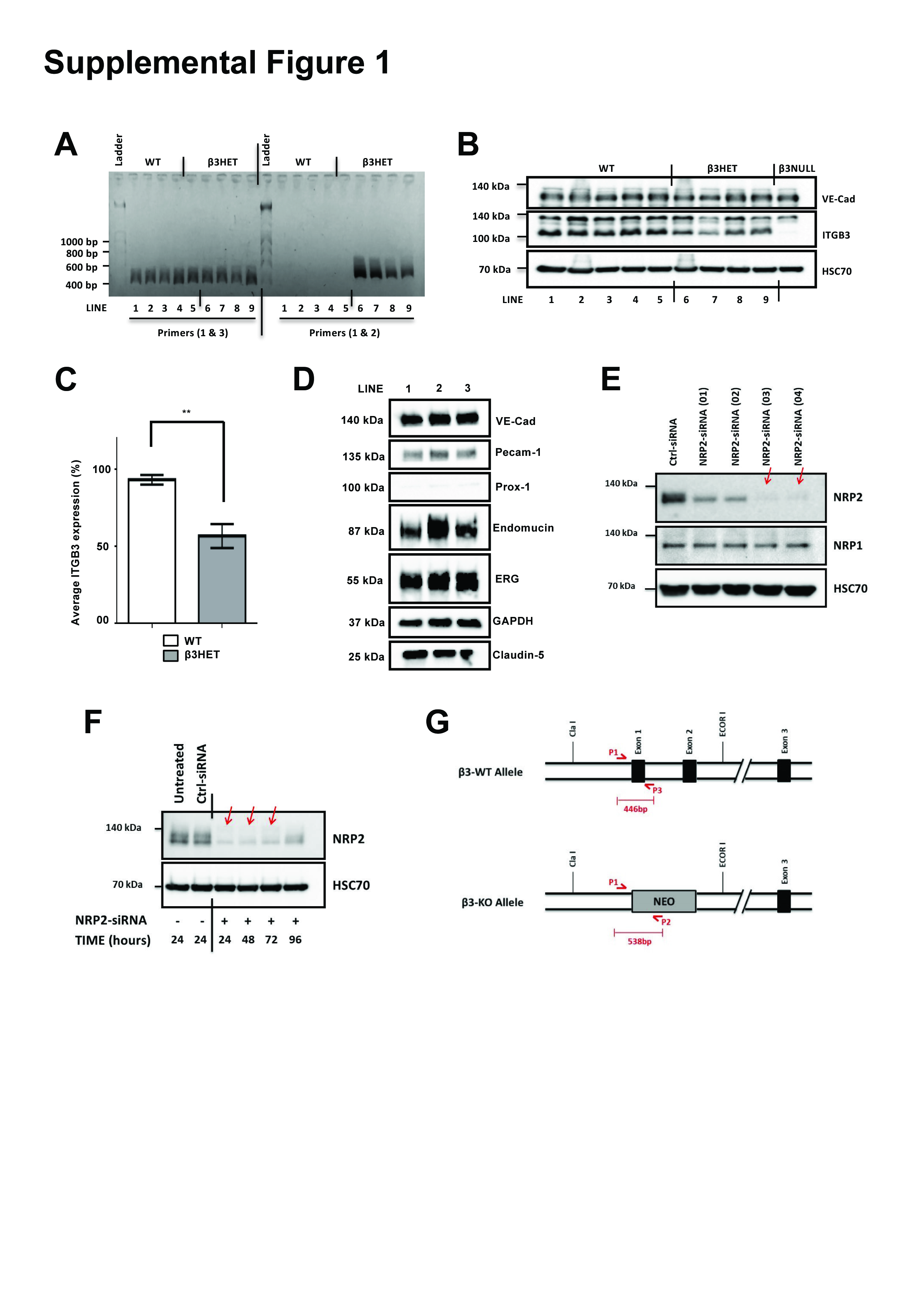

Supplement: FIGURE S1 — (A): PyMT transfected mLMEC pellets from nine different immortalized EC lines were subjected to DNA extraction and PCR-based genotyping at the ITGB3 locus. Shown is an agarose gel of P1/P3 and P1/P2 primer products from all EC lines. Lines 1–5 show only a P1/P3 product and are therefore wild-type (WT) for the ITGB3 locus, whilst lines 6–9 show both a P1/P3 product, and a P1/P2 product, and are therefore heterozygous (β3HET) for the ITGB3 locus. (B): Western blot analysis of VE-Cadherin and ITGB3 expression in the same clones shown in (A). HSC70 was used as a loading control. Because the antibody used for detection of ITGB3 recognizes a non-specific band at approximately (135kDa), a β3-knockout (NULL) lysate was included as a control (cell line #10). (C): Left panel shows the densitometric analysis of mean ITGB3 band intensities normalized against HSC70 obtained using ImageJTM [from the Western blot shown in (B)]. ∗∗P < 0.01. (D): The EC identity of three immortalized EC lines was re-confirmed by Western blot analysis, immunoblotting against additional EC markers Pecam-1, Endomucin, ERG and Claudin-5, alongside a GAPDH loading control and a lymphatic marker Prox-1. (E): ECs were transfected either with control siRNA or one of four different NRP2-specific siRNAs (01–04) and incubated for 48 h. EC extracts were then subjected to Western blot analysis using antibodies against NRP2, NRP1 and HSC70. Except where noted (Supplementary Figure S3), NRP2 siRNA #03 was used for all subsequent experiments to silence NRP2 expression. (F): siRNA-transfected ECs were incubated for the indicated timepoints before being lysed and subjected to Western blot analysis using antibodies against NRP2 and HSC70. Asterisks indicate statistical significance from unpaired two-tailed t-tests. (G): Schematic diagram of the ITGB3 (WT) and (HET) loci, showing where PCR genotyping primers align. P1 and P3 amplify a wild-type product of 446-bp, whilst P1 and P2 amplify a knockout product of 538-bp. [file Image_1.TIF]

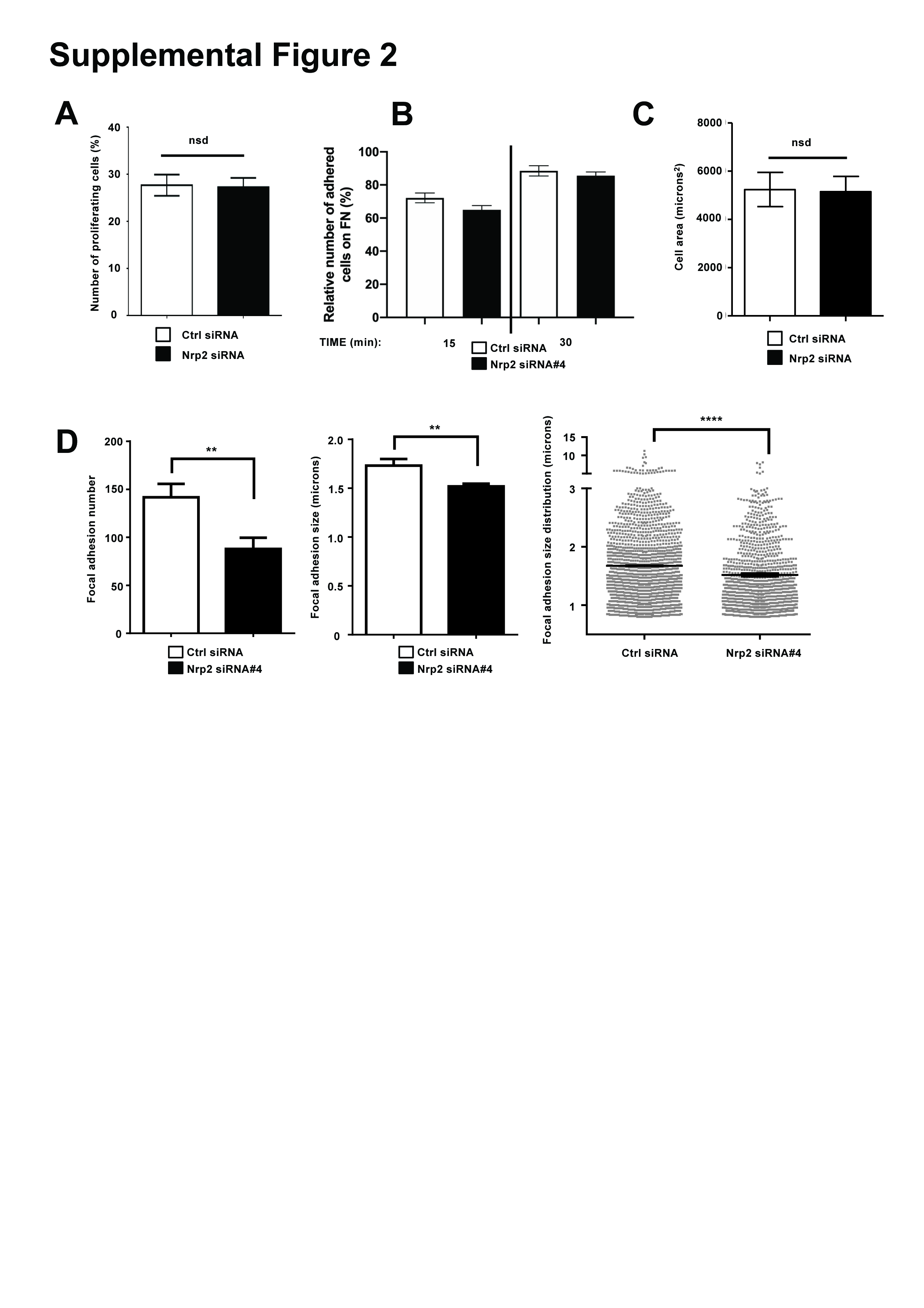

Supplement: FIGURE S2 — (A): siRNA-transfected ECs were seeded onto FN and incubated for 30 h. ECs were then re-seeded onto FN-coated coverslips, and allowed to adhere for 4 h in serum-free media. Media was then replaced with 10 μM BrdU in complete culture medium, and the cells incubated for 12 h at 37°C. ECs were then fixed with 4% PFA, before hydrolyzing the DNA with 1 M HCL. ECs were permeabilized and blocked with Dako® Protein Block Serum-Free. ECs were then incubated with anti-BrdU at 4°C overnight in a humidified chamber, followed by incubation in secondary antibody. Coverslips were subsequently mounted and the number of cells in S-phase (proliferating cells) was determined by dividing the number of the BrdU-labeled cells by the number of DAPI-labeled cells. n = 19 independent fields of view, containing on average 50 cells per field, per condition. (B): Adhesion assay performed as described in Figure 2A, however, ECs were transfected with either control or NRP2 siRNA#04. Bars show mean number of adhered cells calculated from absorbance readings from 40 wells per condition, per timepoint, normalized to a 3-h incubation control plate, N = 1. (C): Accompanying analysis to Figure 2D. The cell area (microns2) was measured using ImageJTM. Quantification performed on mean data from n ≥ 25 ECs over N = 3 independent experiments, nsd = not significantly different from unpaired two-tailed t-test. (D): siRNA-transfected ECs were prepared as described in Figure 2D legend, measuring mean FA number, FA size and FA distribution, however, ECs were transfected with either control or NRP2 siRNA#04. Quantification performed on mean data from n = 15 ECs. Asterisks indicate statistical significance from an unpaired two-tailed t-test. [file Image_2.TIF]

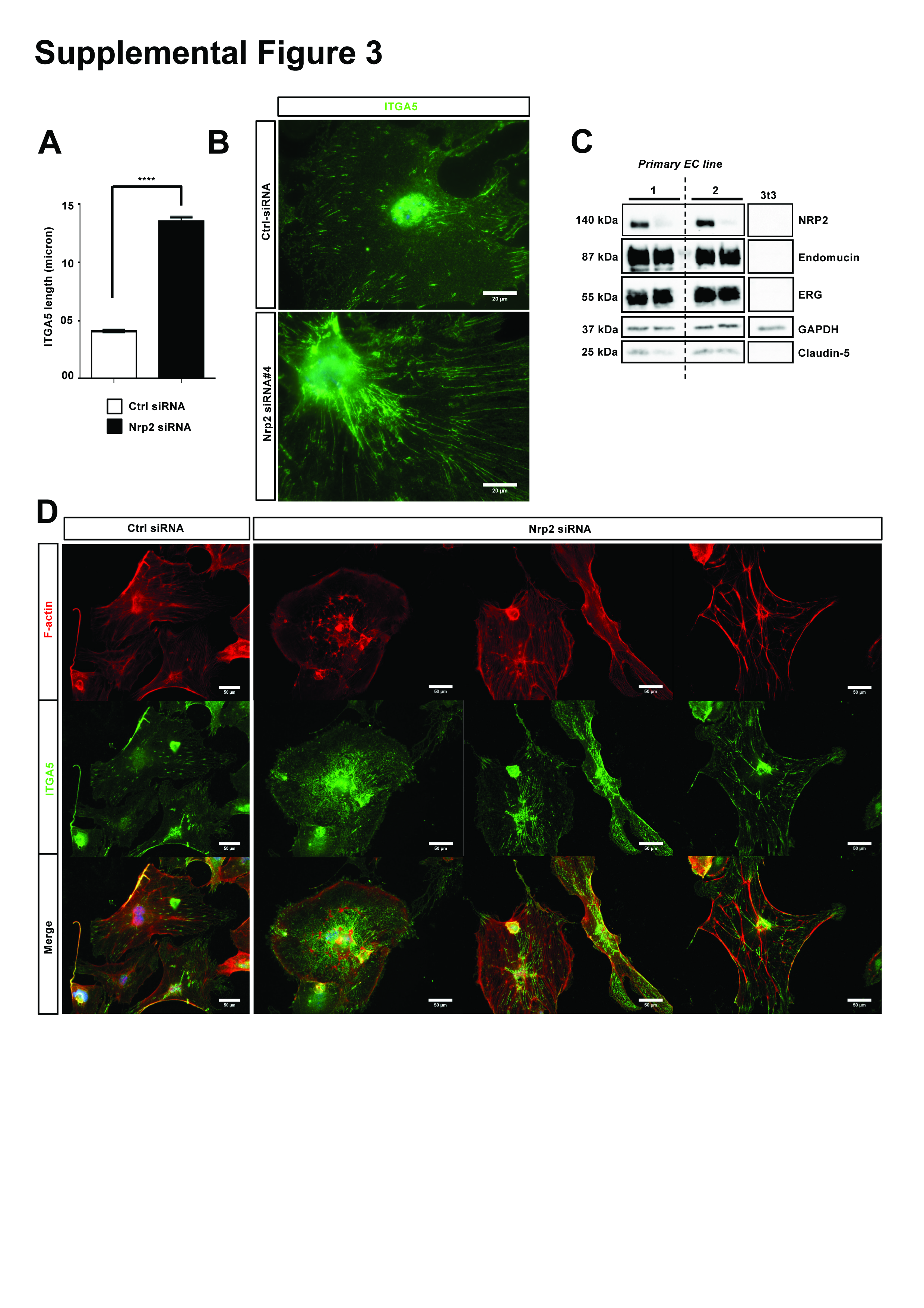

Supplement: FIGURE S3 — (A): Accompanying analysis to Figure 3D. siRNA-transfected ECs were prepared for immunocytochemistry as described in Figure 2C. Fixed ECs were incubated in primary antibodies against ITGA5 overnight at 4°C. ITGA5 length was measured using the ImageJTM software plugin simple neurite tracer. n = 490 cells per condition, ****P (0.0001). Asterisks indicate statistical significance from unpaired two-tailed t-tests. (B): siRNA-transfected ECs from three different immortalized EC lines were prepared as described in Figure 3D legend, however, ECs were transfected with either control (top) or NRP2 siRNA#04 (bottom). Panels show representative images from N = 3 independent lines, n ≥ 10 cells per line. (C): Western blot analysis of cell lysates from both primary EC clones alongside a lysate from a known fibroblast control cell line. EC extracts were immunoblotted using antibodies against known EC markers Endomucin, ERG and Claudin-5, alongside a GAPDH loading control. (D): Primary ECs were transfected with either ctrl or NRP2 siRNA and prepared for immunostaining as described in Figure 3E. Panels show representative images from n = 10 cells per condition from two independent primary EC lines. [file Image_3.TIF]
