# Supplementary material for: NRP2 as an Emerging Angiogenic Player; Promoting Endothelial Cell Adhesion and Migration by Regulating Recycling of α5 Integrin
Source: Front Cell Dev Biol. 2020 May 26;8:395. doi: 10.3389/fcell.2020.00395 (PMC7264094; doi:10.3389/fcell.2020.00395)
Supplement: TABLE S1 — NRP2-immunoprecipitating label-free quantitative (LFQ) mass spectrometry list. [file Table_1.DOCX]

| Table 1: NRP2-immunoprecipitating Label-Free Quantitative (LFQ) mass spectrometry list | | | |
| --- | --- | --- | --- |
| Gene name: | **Hit 1** | **Hit 2** | **Average fold change (Log2LFQ^Ctrl siRNA-NRP2 siRNA^)** |
| Actb;Actg1 | 26.74492 | 27.96095 | 27.352935 |
| Actbl2 | 25.99547 | 27.82845 | 26.91196 |
| Vim | 26.34357 |  | 26.34357 |
| Myh9 |  | 24.38293 | 24.38293 |
| Capza1 |  | 23.21312 | 23.21312 |
| Myh10 |  | 22.79844 | 22.79844 |
| Tpm4 |  | 22.57404 | 22.57404 |
| Tjp1 |  | 22.57389 | 22.57389 |
| Capza2 | 21.93517 | 22.74181 | 22.33849 |
| Pecam1 | 21.15972 | 23.34476 | 22.25224 |
| Tjp2 |  | 22.24607 | 22.24607 |
| Twf1 |  | 22.19763 | 22.19763 |
| Arpc2 |  | 22.09911 | 22.09911 |
| Ckap4 | 21.97439 |  | 21.97439 |
| Arpc4 |  | 21.86602 | 21.86602 |
| Tpm1 |  | 21.71132 | 21.71132 |
| Lima1 |  | 21.59035 | 21.59035 |
| Dbn1 |  | 21.52365 | 21.52365 |
| Tmod3 | 19.75966 | 23.26303 | 21.511345 |
| Capzb |  | 21.04462 | 21.04462 |
| Mcam | 20.20144 | 21.8063 | 21.00387 |
| Dab2 |  | 20.8565 | 20.8565 |
| Arpc1b |  | 20.82736 | 20.82736 |
| Cav1 | 21.52741 | 19.57003 | 20.54872 |
| Cfl1 |  | 20.52987 | 20.52987 |
| Add1 |  | 20.48766 | 20.48766 |
| Myo1c |  | 20.41087 | 20.41087 |
| Rac1 |  | 20.33898 | 20.33898 |
| Map4 |  | 20.29954 | 20.29954 |
| Actr2 |  | 20.16754 | 20.16754 |
| Actg1 | 21.38089 | 18.84251 | 20.1117 |
| Cav2 |  | 20.05977 | 20.05977 |
| Itga6 |  | 20.0482 | 20.0482 |
| Itgb1 | 19.58851 | 20.40907 | 19.99879 |
| Eps15l1 |  | 19.98542 | 19.98542 |
| Ap2m1 |  | 19.94502 | 19.94502 |
| Icam1 |  | 19.91639 | 19.91639 |
| Pcdh1 |  | 19.81745 | 19.81745 |
| Ptrf |  | 19.77045 | 19.77045 |
| Tpm1 |  | 19.5741 | 19.5741 |
| Itga3 | 19.19916 |  | 19.19916 |
| Arpc5l |  | 19.19293 | 19.19293 |
| Ybx3;Igf2bp3 |  | 19.06616 | 19.06616 |
| Snx9 |  | 19.04328 | 19.04328 |
| Col18a1 |  | 18.94963 | 18.94963 |
| Ap2s1 |  | 18.87728 | 18.87728 |
| Plcb4 |  | 18.86502 | 18.86502 |
| Cttn |  | 18.84761 | 18.84761 |
| Actc1;Acta2;Actg2;Acta1 | 16.71621 | 20.74104 | 18.728625 |
| Ablim1 |  | 18.72782 | 18.72782 |
| Actr3 |  | 18.68072 | 18.68072 |
| Itga5 | 17.63318 | 19.69024 | 18.66171 |
| Plec | 18.63159 |  | 18.63159 |
| Cd44 | 18.60012 |  | 18.60012 |
| Msn |  | 18.52815 | 18.52815 |
| Vasp |  | 18.36514 | 18.36514 |
| Cdh13 |  | 18.36345 | 18.36345 |
| Sdpr |  | 18.27245 | 18.27245 |
| Dnm2 |  | 18.22985 | 18.22985 |
| Tuba1b;Tuba4a | 17.77056 | 18.55225 | 18.161405 |
| Arpc5 |  | 18.14685 | 18.14685 |
| Wdr1 |  | 18.12918 | 18.12918 |
| Rala | 17.51902 | 18.64505 | 18.082035 |
| Rab11fip5 |  | 18.07134 | 18.07134 |
| Shank3 | 17.82211 | 18.26145 | 18.04178 |
| Tmed10 | 18.03238 |  | 18.03238 |
| Ralb | 17.80305 | 18.1332 | 17.968125 |
| Mmrn2 |  | 17.95231 | 17.95231 |
| Tmod2 |  | 17.90464 | 17.90464 |
| Ap2b1 |  | 17.88544 | 17.88544 |
| Add3 | 15.54402 | 19.90345 | 17.723735 |
| Rab6a;Rab6b | 17.7023 |  | 17.7023 |
| Rab14 | 17.70072 |  | 17.70072 |
| Ehd4 |  | 17.67049 | 17.67049 |
| Pdcd10 | 17.15686 | 18.14309 | 17.649975 |
| Esam | 17.52096 |  | 17.52096 |
| Myo6 |  | 17.50802 | 17.50802 |
| Emd | 17.48906 |  | 17.48906 |
| Tnc |  | 17.48901 | 17.48901 |
| Pcdh1 |  | 17.46153 | 17.46153 |
| Vapa | 17.45271 |  | 17.45271 |
| Rab18 | 17.37967 |  | 17.37967 |
| Cdc42ep1 |  | 17.34617 | 17.34617 |
| Flna |  | 17.30314 | 17.30314 |
| Cct4 | 17.25002 |  | 17.25002 |
| Vti1b |  | 17.2326 | 17.2326 |
| Bcam |  | 17.20623 | 17.20623 |
| Tmem2 |  | 17.13397 | 17.13397 |
| Picalm |  | 17.13025 | 17.13025 |
| Vamp7 |  | 17.12582 | 17.12582 |
| Adam9 |  | 17.1037 | 17.1037 |
| Stx6 |  | 17.08659 | 17.08659 |
| Rab35 |  | 17.0683 | 17.0683 |
| Gm17087 | 17.0524 |  | 17.0524 |
| Tpm1 |  | 17.04772 | 17.04772 |
| Scamp3;Tu52 | 16.18946 | 17.89125 | 17.040355 |
| Rab7;Rab7a | 17.02443 |  | 17.02443 |
| Triobp |  | 17.00441 | 17.00441 |
| Synpo |  | 16.99803 | 16.99803 |
| Vamp3;Vamp2 |  | 16.97365 | 16.97365 |
| Myo5a |  | 16.95829 | 16.95829 |
| Myl12a |  | 16.95068 | 16.95068 |
| Twf2 | 16.10847 | 17.78743 | 16.94795 |
| Ppp1r9b |  | 16.89936 | 16.89936 |
| Cdh5 |  | 16.8382 | 16.8382 |
| Ldlr | 16.65608 | 17.01857 | 16.837325 |
| Rras |  | 16.78979 | 16.78979 |
| Sdcbp | 16.56674 | 17.01283 | 16.789785 |
| Ehd1 | 16.74583 |  | 16.74583 |
| Palm |  | 16.74272 | 16.74272 |
| Pdlim7 | 16.62921 |  | 16.62921 |
| Sec22b | 16.56748 |  | 16.56748 |
| Tpm1 |  | 16.56535 | 16.56535 |
| Itgb3 |  | 16.55691 | 16.55691 |
| Epn2 |  | 16.55035 | 16.55035 |
| Arpc3 |  | 16.54422 | 16.54422 |
| Marcksl1 |  | 16.49389 | 16.49389 |
| Grb2 |  | 16.43816 | 16.43816 |
| Scamp2 |  | 16.37087 | 16.37087 |
| Iqgap1 |  | 16.32632 | 16.32632 |
| Rab5c | 16.27054 |  | 16.27054 |
| Ap2a1 | 15.59941 | 16.89696 | 16.248185 |
| Flot1 |  | 16.2365 | 16.2365 |
| Epn1 |  | 16.21974 | 16.21974 |
| Epha2 | 16.21529 |  | 16.21529 |
| Ktn1 | 16.17446 |  | 16.17446 |
| Fscn1 |  | 16.16555 | 16.16555 |
| Ap2a2 |  | 16.04031 | 16.04031 |
| Actn4 | 15.90556 |  | 15.90556 |
| Marcks |  | 15.88345 | 15.88345 |
| Add2 | 13.89125 | 17.71187 | 15.80156 |
| Rhoc;Rhoa;Rhob | 15.73653 |  | 15.73653 |
| Ptk7 |  | 15.70356 | 15.70356 |
| Tubb5;Tubb2b;Tubb2a;Tubb3 | 16.61827 | 14.75881 | 15.68854 |
| Mapre1 |  | 15.64087 | 15.64087 |
| Actr1a | 15.16257 | 15.92509 | 15.54383 |
| Eps15 | 13.49852 | 17.56639 | 15.532455 |
| Rdx |  | 15.48895 | 15.48895 |
| Clta |  | 15.45988 | 15.45988 |
| Cltc;mKIAA0034 | 15.11689 | 15.77486 | 15.445875 |
| Ctnnd1 |  | 15.38476 | 15.38476 |
| Ehd2 | 16.04132 | 14.69171 | 15.366515 |
| Flot2 |  | 15.32638 | 15.32638 |
| Specc1l |  | 15.30421 | 15.30421 |
| Clint1 |  | 15.28675 | 15.28675 |
| Eng | 13.63594 | 16.6332 | 15.13457 |
| Itgav | 12.75036 | 17.41807 | 15.084215 |
| Scamp1 | 14.12918 | 16.03603 | 15.082605 |
| Sh3gl1 |  | 15.05388 | 15.05388 |
| Ctnna1 | 14.46956 | 15.58295 | 15.026255 |
| Ctnnb1 | 14.04132 | 16.01054 | 15.02593 |
| Rap1b |  | 14.92447 | 14.92447 |
| Htra1 |  | 14.8882 | 14.8882 |
| Vps13b |  | 14.86762 | 14.86762 |
| Map1b |  | 14.83246 | 14.83246 |
| Sdc3 |  | 14.67728 | 14.67728 |
| Golim4 | 14.59475 |  | 14.59475 |
| Rab1b | 14.23007 |  | 14.23007 |
| Icam2 | 13.61156 |  | 13.61156 |
| Grasp |  | 13.39968 | 13.39968 |
| Cope | 12.94733 | 13.11721 | 13.03227 |
| Cct3 | 12.96404 |  | 12.96404 |
| Notch1 |  | 12.79969 | 12.79969 |
| Golga7 |  | 12.51175 | 12.51175 |
| Smtn |  | 12.48062 | 12.48062 |
| Plvap | 8.196397 | 15.66909 | 11.9327435 |
| Myo18a |  | 11.90463 | 11.90463 |
